# The Cumberland Infirmary, Carlisle

**Published:** 1914-10-24

**Authors:** 


### The Hospital

The Workers' Newspaper of Administrative Medicine and Institutional Life, Administration, National Insurance and Health.

No. 1479, Vol. LVII. SATURDAY, OCTOBER 24, 1914. PRICE ONE PENNY.

### THE CUMBERLAND INFIRMARY, CARLISLE.

This infirmary has recently been reconstructed and enlarged, so that the accommodation for in-patients has been increased from 100 to 150 beds. The far-reaching character and extent of these additions and alterations may be realised from the Report of Sir Henry Burdett, which we published on September 19, 1914. Their importance is confirmed by the fact that the sum expended was, in round figures, £30,000. To Dr. Henry Barnes, consulting physician to the infirmary, who was chairman of the committee in 1909, the governors and inhabitants of Carlisle and the district served by the infirmary owe life-long gratitude and thanks. If it had not been for Dr. Barnes' whole-hearted interest in and affection for this hospital, it is probable that the accommodation now provided and the possibilities which it at present affords to the committee to bring its administration up to date and to extend its usefulness would not exist to-day. Indeed, if we may judge by the letter from Dr. Lediard in the *Lancet* of the 17th inst., referred to on another page, he and presumably others amongst the governors were opposed to portions of the scheme of reconstruction which were undoubtedly called for in the best interests of an institution which maintains a training school for nurses. Any opposition of the kind, if it exists, can only emphasise the immense service which Dr. Barnes and his supporters have rendered to the infirmary by their generous liberality and courage in proceeding with the reconstruction and completing it in its entirety.

The Cumberland Infirmary being now brought up to date structurally, it is to be hoped that its system of administration will be thoroughly overhauled so as to take full advantage of the facilities offered by the reconstruction to make every department efficient. During the last twenty years, in consequence of the vast changes and increased cost of maintaining each voluntary hospital at the highest possible standard, facilities have been afforded whereby the adequate training of all who wish to qualify themselves for the higher appointments in the hospitals has been effected. It has been demonstrated over and over again that economy in the management, the raising of an adequate income, the establishment and maintenance of an equitable system of control (whereby the rights and happiness of every section of the resident staff and the independence of the head of each department are continuously secured) entail the appointment of none but experienced, trained, and knowledgeable men and women to fill the higher posts at our voluntary hospitals. Where these patent facts are recognised and acted upon, efficient administration and financial prosperity prevail. It has been evident that to point out and insist upon a change of system in the administration of a voluntary hospital is apt to be resisted by the managers on the ground that any such change must cast a slur upon the committee. This view arises often from the failure of a committee to apprehend that hospital administration is a progressive science, and that when reconstruction takes place the area and volume of work, and with them the need for greater monetary support, increase too. To meet these extra demands successfully, and to make an old foundation renew its life and extend its usefulness to the full, new methods of administration, with skilled and trained officers of the highest obtainable quality, often become essential. In such cases a change in the system of administration, when agreed to by the committee of management, constitutes the latter's highest claim to public confidence and recognition. It can never be a reflection upon, but must always in fact be convincing evidence of, the efficiency and public spirit of every committee which pursues such a policy. In the older institutions, where officials may have held office for years and have become wedded to old methods and old ways which cause them the minimum of work and trouble, experience has shown that the most equitable and wisest course for a committee to take is to remove any possibility of obstruction by voting a pension or two. The economy of such a course is proved by precedents innumerable.

The report of the Cumberland Infirmary and the correspondence which has arisen upon it, which appeared in The Hospital of September 19 and October 10, 1914, are at present exciting interest and discussion in Carlisle and the district around. The *Cumberland News*, in an excellent leading article, deals with the matter from some local points of view. The *Cumberland News*

appears to be unaware that what Dr. Lediard called "certain inaccuracies in Sir Henry's Report which mar the trustworthiness of his statements" have been shown not to be inaccuracies of Sir Henry Burdett. That Report calls for a change of system in the administration, and we have shown that this suggestion casts no reflection, as our contemporary considers, upon the committee of management. The *Cumberland News* claims that the raising of £30,000 and its expenditure on reconstruction prove that "spirit, interest, and life" are not wanting locally, and that it is unjust and undeserved to say that they are. Our contemporary announces "the cool reception of Sir Henry's Report" by the "public," but how can this be, seeing that no local paper appears to have published the Report, and the public is therefore probably ignorant of its existence? But the principal plea upon which the *Cumberland News* rests its defence of inaction is that a change of system will mean an extra £1,000 a year of expenditure. There will be no necessity for a Fortunatus's purse, however, providing the reform of system is wisely and firmly adopted and enforced, as an inquiry on the lines suggested in the Report would, we are convinced, prove. That is one reason why we hold the opinion that a special investigation and an independent report by a hospital expert of wide experience are called for.

